# Research influence on antimalarial drug policy change in Tanzania: case study of replacing chloroquine with sulfadoxine-pyrimethamine as the first-line drug

**DOI:** 10.1186/1475-2875-4-51

**Published:** 2005-10-20

**Authors:** Godfrey M Mubyazi, Miguel A Gonzalez-Block

**Affiliations:** 1 National Institute For Medical Research (NIMR), Department of Health Systems and Policy Research, Centre for Enhancement of Effective Malaria Interventions (CEEMI), P.O Box 9653, Dar es Salaam, United Republic of Tanzania; 2 Alliance For Health Policy and Systems Research (AHPSR), World Health Organization, Geneva, Switzerland

## Abstract

**Introduction:**

Research is an essential tool in facing the challenges of scaling up interventions and improving access to services. As in many other countries, the translation of research evidence into drug policy action in Tanzania is often constrained by poor communication between researchers and policy decision-makers, individual perceptions or attitudes towards the drug and hesitation by some policy decision-makers to approve change when they anticipate possible undesirable repercussions should the policy change as proposed. Internationally, literature on the role of researchers on national antimalarial drug policy change is limited.

**Objectives:**

To describe the (a) role of researchers in producing evidence that influenced the Tanzanian government replace chloroquine (CQ) with sulfadoxine-pyrimethamine (SP) as the first-line drug and the challenges faced in convincing policy-makers, general practitioners, pharmaceutical industry and the general public on the need for change (b) challenges ahead before a new drug combination treatment policy is introduced in Tanzania.

**Methods:**

In-depth interviews were held with national-level policy-makers, malaria control programme managers, pharmaceutical officers, general medical practitioners, medical research library and publications officers, university academicians, heads of medical research institutions and district and regional medical officers. Additional data were obtained through a review of malaria drug policy documents and participant observations were also done.

**Results:**

In year 2001, the Tanzanian Government officially changed its malaria treatment policy guidelines whereby CQ – the first-line drug for a long time was replaced with SP. This policy decision was supported by research evidence indicating parasite resistance to CQ and clinical CQ treatment failure rates to have reached intolerable levels as compared to SP and amodiaquine (AQ). Research also indicated that since SP was also facing rising resistance trend, the need for a more effective drug was indispensable but for an interim 5–10 year period it was justifiable to recommend SP that was relatively more cost-effective than CQ and AQ. The government launched the policy change considering that studies (ethically approved by the Ministry of Health) on therapeutic efficacy and cost-effectiveness of artemisinin drug combination therapies were underway. Nevertheless, the process of communicating research results and recommendations to policy-making authorities involved critical debates between policy makers and researchers, among the researchers themselves and between the researchers and general practitioners, the speculative media reports on SP side-effects and reservations by the general public concerning the rationale for policy change, when to change, and to which drug of choice.

**Conclusion:**

Changing national drug policy will remain a sensitive issue that cannot be done overnight. However, to ensure that research findings are recognised and the recommendations emanating from such findings are effectively utilized, a systematic involvement of all the key stakeholders (including policy-makers, drug manufacturers, media, practitioners and the general public) at all stages of research is crucial. It also matters how and when research information is communicated to the stakeholders. Professional organizations such as the East African Network on Malaria Treatment have potential to bring together malaria researchers, policy-makers and other stakeholders in the research-to-drug policy change interface.

## Introduction

Malaria is still the leading cause of morbidity and mortality in sub-Saharan Africa especially in young children and pregnant women [1]. Considering the limited health budgets and the rising cost of medical services, the increasing trends of drug resistance raise critical public health concerns, as this constrains the provision of adequate treatment in countries where the disease is endemic. The increasing evidence on *Plasmodium falciparum *parasite resistance to chloroquine (CQ) has prompted some countries to revise their treatment guidelines [2]. In the last decade, the immediately considered alternative first-line drug in some southern African countries, such as South Africa, Botswana, Malawi and Kenya, was SP, but now, countries such as Burundi and Rwanda have already opted for artemisinin drug combination therapy while several others are considering to do so [3]. In many countries, the hesitation by ministries of health to make a policy change decision has been over-influenced by economic budget considerations [4,5]. In Tanzania, the critical nature of the decision to switch to a new first-line drug was closely linked with the estimated budget for the policy change of US $100.8 million [6], which represented 3.4% of the GNP.

Located between latitudes 1°S and 12°S and longitudes 30°E and 40°E, the United Republic of Tanzania covers an area of 945,050 km^2^, including 59,050 km^2 ^of inland waters [7]. Malaria has been the most life-threatening public disease in terms of morbidity and mortality in Tanzania since the colonial era, and today has a critical influence on the poverty cycle. One recent study showed that malaria reduces the national economic growth by 1.3% [8]. The same study revealed that contributing to about 40,000 deaths annually malaria puts nearly 35 million Tanzanian population at risk. Furthermore, records show malaria accounting to a third of all outpatient visits, a third of inpatient admissions and a third of deaths among children under the age of five years admitted to hospitals [9,10]. Also the national annual health-facility-based statistics for the last ten years (1995–2004) indicate malaria as the highest cause of outpatient attendance in people aged 5 years and above and deaths in hospitalized patients of all age groups.

In Tanzania, the capacity to respond to this malaria problem and other health crises is highly constrained by the meagre Government spending of US $4 per capita on health, with a range of 8–10% of total government budget [11]. The large external debt exerts pressure on the Government's limited resources and in its struggle to eradicate poverty. Tanzania is one of the poorest countries with a per capita income of US $170 per year, about 27% of the population spending less than $0.50 per day on overall needs, 48% spending less than $0.65 per day on basic needs while the real annual economic growth-rate was about 4.9% by year 1999 [12].

For the last 45 years or so, CQ has been the first line drug in nearly all malaria endemic SSA countries. This is due to its ready availability in kiosks, shops and drug stores, as well as from formal health facilities, its relatively low cost per dose and its safety [13]. Until the last day of July 2001, CQ was officially the first-line drug for the treatment of uncomplicated malaria in Tanzania, SP being the second-line drug while quinine was the third-line drug for severe or complicated malaria [14]. As in many other tropical countries, the treatment of malaria has ranged from self-medication using traditional medicines to the use of modern pharmaceuticals [15]. In the context of high levels of poverty, exacerbated by diseases and external pressures, it is initially difficult to implement large-scale and sustainable policy changes even if there is available evidence to support them. This has been the case with recent changes in the malaria treatment policy in Tanzania.

## Conceptual framework

The paper is part of a series of case-studies undertaken by the Alliance for Health Policy and Systems Research (AHPSR) to identify the production and utilization of research evidence for policy making in developing countries. An attempt has been made to describe an enabling environment for the demand for research evidence and its utilization, including research funding, research priority setting, institutional support and commissioning. Case-studies were prepared to encourage discussion about the processes and mechanisms which affect support for research and its impact and identification of the challenges in setting research priorities, decision-makers' support for research and the benefits gained from the research process and its results.

In the Tanzanian case-study, particular attention was paid to describing the role of diverse mechanisms and actors in the research-to-policy change process. The impact of HPSR was analysed by observing research inputs and decision outputs in specific policy development situations [16]. Research inputs would be studied from the supply side by analysing problems of HPSR dissemination, and from the demand side through an examination of the participation of researchers as part of the policy-making process. The influence of different types of knowledge – from empirical findings in data-driven design situations to broad conceptual policy frameworks was explored.

**Figure 1:**
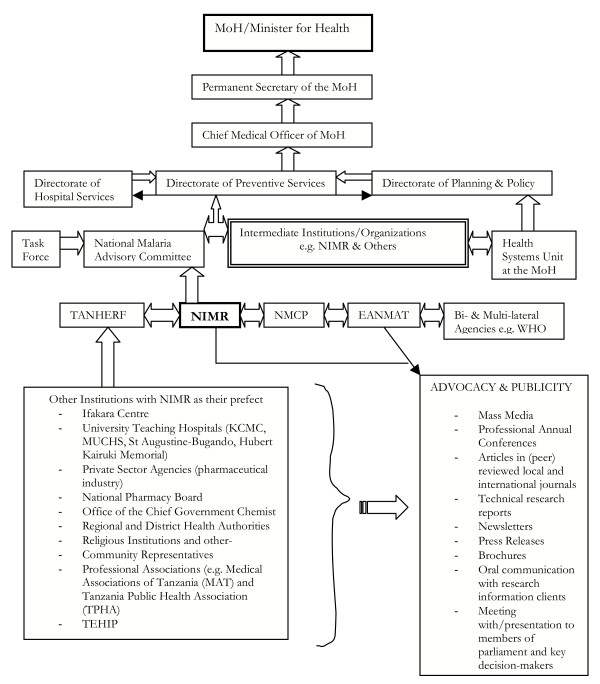
Actors in the research-to-policy interaction process in Tanzania.

**Figure 2:**
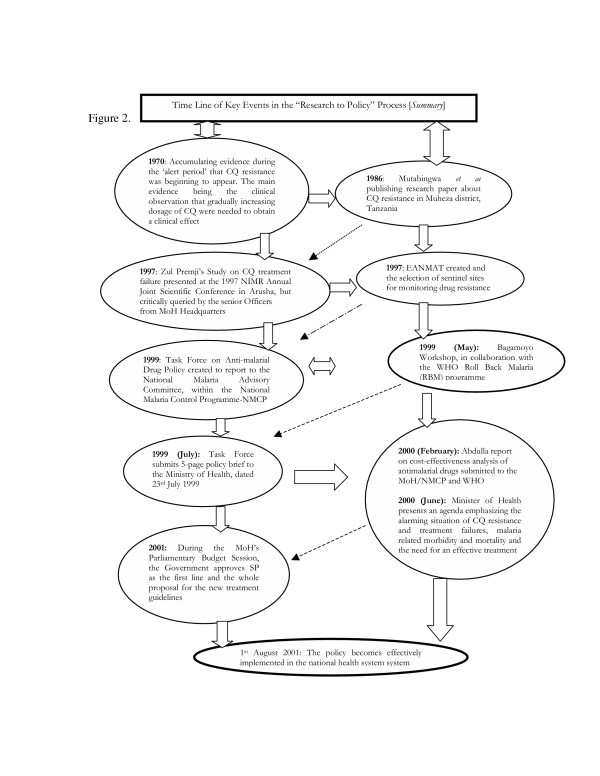
Time-line of key events in the Research-to-Policy process (summary).

Research is greatly under-utilized as a tool to guide health policy formulation, improvement and practice, particularly in the challenges of scaling up interventions and improving access to services in developing health systems. There is a general lack of formal interfaces and linkage strategies to ensure that research supports policy development. To improve utilization, it is important to reveal the policy framework through which researchers can or have influenced decisions. This involves looking at how policy-makers and service managers can use or have effectively used the available research information and how researchers can be or have been promoted to interact among themselves and other key stakeholders in the policy arena [16]. In Tanzania and East Africa, a number of policy initiatives and research projects have supported research undertakings, evidence production and utilization. The Tanzania National Health Research Forum (TANHERF) has been the prime priority-setting mechanism since 1999 and has led to the establishment of an institutional framework for mediating the communication of research evidence to policy-makers [17]. The NIMR and TEHIP have functioned as secretariat for the Forum [18,19] (see Figures 1 &2).

In this paper, a description is given on the process of changing the malaria treatment policy by replacing CQ with SP as the first-line antimalarial drug in Tanzania. Focus is on the process and role of researchers in providing evidence to and their interaction with policy-makers and other stakeholders and the challenges ahead before a government decision to switch from SP to artemisinin drug combination therapy.

## Study Methods

### Scope of the study and study design

The case study was exploratory in design, undertaken between June, 2001 and November, 2002 to assess the value of existing institutional mechanisms and interfaces in Tanzania with reference to a recent national antimalarial drug policy change. Focus has been on the role of, and methods used by researchers and research institutions in producing and communicating evidence on antimalarial drug resistance situation and cost-effectiveness of alternative drugs to policy-makers and other stakeholders (medical practitioners, local pharmaceutical manufacturers, traders and community representatives). The study was built on the conception that research is greatly under-utilized as a tool to guide the health policy-making process, policy improvement and practice, considering the emerging challenges to scaling up interventions and improving access to services in developing health systems of countries such as Tanzania. Also considering that policy-making is greatly influenced by health professionals, industry and to some extent the public [16] it was imperative to analyse the relationship between research undertakings to produce evidence for explicit policy decision and the actual utilization of evidence by policy decision-makers.

### Sampling

The target was to approach people who played one or several of the following roles or by virtue of their designations: research experience on malaria drugs for at least 5 years, members of various committees involved in discussions on drug policy issues at national level, general practitioners, and lecturers in health policy aspects at high learning institutions. In total, 21 officers were interviewed, including 10 senior malaria researchers, two policy-makers, one officer working with the national pharmacy board, four national-level malaria programme managers, five general medical practitioners (of who one was district- and one a regional medical officer), two university teaching hospital lecturers, two medical research publications and documentations officers, two heads of medical research institutions. Of all the respondents, eight were medical doctors with long experience in malaria case management.

### Major themes covered in the indepth interviews and document review

Generally interviewees were asked for their opinions about (i) initiatives that were available to produce research evidence to inform national policy-makers about the efficacy and effectiveness of different antimalarial drugs, the actors involved in such research initiatives and the financial, institutional and political environment to support such initiatives in Tanzania. This component looked at what prompted researchers develop interest in antimalarial drug studies (including how the research topic was identified and priorities set), and how were the studies funded and how were the reports from such studies communicated to policy-makers and other potential stakeholders (ii) research reports showing that due to increasing trend in parasite resistance to CQ and its treatment failures compared to other antimalarial drugs the government urgently needed to revise its malaria treatment guidelines (iii) as potential research-to-policy actors, experiences they have had seeing research evidence utilised by national antimalarial drug policy-makers. This component observed the mechanisms involved in bringing together researchers and policy-makers to policy discussion tables on aspects related to malaria treatment using various antimalarial drugs and the challenges faced so far and those ahead. The document review complemented the interviews by observing the local and international institutions involved in the antimalarial research-to-policy interplay since independence, the funding of the research, the output from several studies commissioned by the government, the WHO and bilateral and multilateral organisations supporting health research and development.

### Data collection methods

In 2001, WHO Geneva through technical experts of the Alliance for Health Policy and Systems Research (AHPSR), an initiative of the WHO and the GFHR provided a standard study framework highlighting key themes to be covered by all the case studied supported by the AHPSR. The framework was validated for its applicability in the context of the country and topic of the case study in question. It also provided guidance for the analysis of the study results. Based on such a framework, research instruments were designed to allow the collection of information through (i) a review of official reports and other documents (ii) in-depth interviews with malaria researchers, policy-makers, programme managers and health workers and at institutional and national levels and the national pharmacy board officers (iii) participant observation (the listed first author in this paper participated in the study in the costs, effects and cost-effectiveness of changing the first-line antimalarial drug policy in Tanzania, a study which was commissioned by the NMCP and undertaken by researchers from four research institutions as identified later. Additional information was obtained from district and regional medical officers' meetings, participation in health research and policy conferences held in the country as organized by NIMR, TMA, TPHA and the Multilateral Initiative on Malaria.

### Data handing and analysis

Most of the respondents suggested to be confronted as many times as it would be found necessary for additional information or clarity needed, so did not see the need for being tape-recorded. Therefore, interviews were recorded through hand written notes and the interviewer was keen in noting down all the important explanations expressed by the individual respondents. With reference to the study themes/questions and as is recommended in case studies [20], the analysis exercise looked at the content, context, actors, process and pattern of the views expressed by different individual respondents. Attempt was made to note the similarities and contrasts and the possible explanations for the contrasting views.

## Results

A summary of the content, patterns, context, actors and processes involved in the research-to-policy process has been presented in Table 2. The subsequent sections give a more detailed account of the interface and interplay between and among the actors involved in the process.

**Table 2:** Factors facilitating and those hindering the production, dissemination and utilisation of research evidence to guide policy formulation process

**Factor**	**Barrier Factors**	**Facilitating Factors**
*Contextual*	-Poverty: (i) Possibility that the drug proposed to replace the existing one may not be cost-effective given the poverty situation facing the majority of the residents (ii) Resource poor government – meagre health budget, high national debt crisis	-UN agencies e.g. WHO and bilateral agencies e.g. DFID, SDC, USAID, DANIDA etc. readiness to assist technically and financially -Other countries in the Region also changing their national treatment policy
*Actors/Institutional*	-Fear by drug manufacturers and traders mainly when they still have huge stocks of the drug proposed to be replaced -Perceptions by doctors/clinicians based on their experiences with prescription/use of alternative antimalarial drugs -Perceptions of some biomedical researchers and national level policy decision makers Sometimes contrasting/overlapping research evidence about drug resistance and cure rates of various drugs (lack of/delayed consensus) -Anticipated repercussions about (i) drug's side effects (ii) poor compliance by drug users and sometimes by drug administrators -Sustainability in government health budget should donors pull out/terminate assistance or when external assistance is not guaranteed	-Involvement of key stakeholders in research -Formation and operation of credible Regional organizations such as EANMAT -Presence formal interface between researchers and policy-makers i.e. institutional and policy frameworks such as TANHERF and professional associations such as national Drug Task Policy Force, MAT, TPHA, and NMAC -Strong local and biomedical research capacity supported by Northern Institutions, bilateral organisations such as DANIDA, DFID, SDC, USAID and multilateral agencies such as TDR
*Content*	-Cost of alternative drugs -Cost of implementing national policy change -Some studies carried out on too small scale in terms of population sample size and area coverage to justify representation of the national picture -Delay in reporting/disseminating research evidence -Delay in policy-makers to make informed decisions based on research evidence and recommendations -Poor communication of research evidence: some reports being too long, some being too technically/professionally written	-Availability of local research evidence on drug resistance -Detailed research reports (i) e.g. Abdulla et al. [1] on cost-effectiveness analysis of alternative treatment policy options and Research synthesis (ii) e.g. brief reports to feedback policy-makers

### Formulation of the need for policy change

The period from mid 1980s marked an increasing interest in antimalarial drug resistance among biomedical researchers in Tanzania. Thus, several small-scale clinical trials were conducted by researchers from the NIMR in collaboration with local and foreign universities, funded by bilateral agencies such as SDC, DANIDA, USAID, DFID and multilateral agencies including WHO, UNDP and UNICEF. Findings from each of such studies gave evidence that CQ was increasingly facing resistance and treatment failures.

### Spread of drug resistance and recommended level to prompt policy change

The need for changing the policy came from accumulated evidence and increasing debates on increasing trend of P. falciparum resistance to CQ, that has been observed in different parts of the country [21-26] since the mid-1950s (also see Table 1 & Figure 1), through to 1970s [27] and the last two decades [4,28]. The Minister of Health, Hon. Anna Abdallah, informed the Parliament of the United Republic of Tanzania during the 2002–2003 budgetary session that her Ministry's decision to suspend CQ as the first-line drug was based on sound evidence pointing to the high cure-rate failure of about 60% while the SP cure rate was 85–90% and was more cost-effective than other antimalarials [27]. While WHO recommends policy change to an alternative drug when the treatment failure reached 25%, evidence from different sentinel sites in the country indicated that up to the time of policy change, CQ treatment failure rate [6] had already reached 52% (ranging 28–72%), 9.5% for SP (ranging 6–32%), and other drugs such as amodiaquine (AQ) and quinine was less than 4.6% (ranging 3.5% – 6%). The increasing numbers of malaria morbidity and mortality in the country, from year to year, was associated by local and foreign researchers with the increasing trend of parasite resistance to CQ. Concern was, therefore, raised about the need to review and improve the national malaria treatment policy guidelines. Another important reason for the increased enthusiasm for policy change towards the end of 1990s is that some countries such as Kenya, Botswana, Malawi and South Africa had already revised their national drug policy guidelines whereby SP had replaced CQ as the first-line drug [6].

**Table 1:** Trends in antimalarial drug resistance in Tanzania, 1950s–1990s

**Period**	**Resistance pattern to chloroquine up to 1999**
Early 1950s	A dose of 2.5 mg/kg is still efficacious (grace period)
Mid-1970s	Owing to slow increase in resistance, the therapeutic dose was gradually increased to the maximum safe level of 25 mg/kg (alert period)
Late 1980s	Resistance to the maximum dose began to reach levels of significant public health importance (alert period)
Mid-1990s	WHO recommended drug policy action if resistance (total treatment failure) reaches 25% (change period)
Late 1990s	Tanzania established sentinel sites in nine regions to monitor resistance by standard methods

Source: Ministry of Health, 1999.

### Actors in research to policy

#### WHO Geneva [through TDR]

Between April–May 1996, WHO through TDR organized an inter-country workshop in Tanga Region, Tanzania aimed at training biomedical researchers and discussing the improved protocol for testing the therapeutic efficacy of CQ, SP and other antimalarials. This workshop was organized to support enhancement of research capacity in collecting, documenting and reporting evidence on antimalarial drug resistance as a milestone for guiding national drug policy review process.

#### EANMAT

Formed in 1997 and supported by DFID, DANIDA and several other agencies, the East African Network on Monitoring Antimalarial Treatment (EANMAT) was established to bring malaria researchers and policy-makers from Ministries of Health in the three East African countries, Kenya, Uganda and Tanzania although Rwanda joined the Network later in 1999. EANMAT's mission is to have a network that would enable the regular monitoring of treatment outcome of the commonly used first and second-line anti-malarial drugs, based on which rational anti-malarial treatment policies would be developed. EANMAT has a secretariat coordinating the activities of member countries according to its constitution and has generated important malaria treatment database that has contributed to the review and modifications of malaria treatment policies in member countries, whereby in Tanzania the data started to be effectively utilized at policy dialogue level in May 1999. EANMAT also provided funds for the second study phase on the efficacy of AQ, Lapdap and other alternative anti-malarial drugs and produced a standard malaria treatment protocol for East African countries for consideration by countries [4].

### Sentinel sites for antimalarial drug resistance monitoring

In 1997, the MoH of Tanzania through its NMCP, in collaboration with EANMAT, decided to select nine areas in different parts of the country to act as sentinel sites for monitoring antimalarial drug resistance. These sites were selected on a number of criteria, including areas with different socio-economic characteristics and accessibility to antimalarial drug sources and varying malaria endemicity and drug resistance patterns. This was followed by the NMCP with support from WHO and other donors to commission a number of studies from research institutions like NIMR and IHRDC, university teaching hospitals, and other institutions to monitor drug resistance and report findings to the Government for consideration. From their inception, the sentinel sites have provided an environment for producing evidence based on which the National Task Force on Antimalarial Drug Policy and EANMAT developed a policy-brief summary to feedback to the national policy makers, who were finally convinced of the widespread CQ resistance and clinical treatment failures throughout the country, which had never happened previously.

### The National Task Force on Antimalarial Drug Policy

The National Task Force on Antimalarial Drug Policy was formulated in May 1999 as a sub-committee of the National Malaria Advisory Committee (NMAC) in consultation with the EANMAT and WHO country office in Dar Es Salaam. This body is comprised of interdisciplinary professionals, some of them from the MoH acting as key policy decision-makers (including the Director of Preventive Services, NIMR's Director General, Muhimbili National Hospital, the National Pharmacy Board, Integrated Management of Childhood Illnesses (IMCI) and the Medical Stores Department). WHO Country office also has been participating in several activities of the Task Force and the NMAC and in providing the necessary and feasible technical and material assistance.

On 23^rd ^July 1999, the Task Force developed a three-page summary, drawing on evidence from clinical trials in the sentinel sites. This information was supplemented with a review of national health management information (HMIS) records, as a research policy-brief to highlight to national policy-makers the trend in antimalarial drug resistance, the status of malaria-related morbidity and mortality, and provide immediate suggestions for short and long term intervention towards effective and sustainable malaria control in the country [10]. The evidence presented in such a brief document was exactly the same as it appeared in the technical research report by Abdulla *et al *[6] warning of the increase of resistance to chloroquine. On that ground, it was recommended that SP should be adopted as the first-line drug in the interim period, while efforts to find the most suitable alternative were underway. It was recommended that the decision to change the policy should be interim because of the increasing evidence on high SP resistance in various parts such as Muheza and Kilombero districts [7]. By that time, neighbouring countries like Malawi and Kenya, as well as South Africa and Botswana had already switched to SP as their first-line drug in 1992 and 1996 respectively [4,24], while records on SP resistance in Malawi indicating to have had remained below 10% over the past six years [6].

### Multi-centre collaborative study on cost-effectiveness of CQ, SP and AQ

In the same year 1999, shortly before the Task Force presented a summary policy brief paper, a consultancy contract was commissioned by the NMCP and WHO-AFRO to NIMR, IHRDC and LSHTM to undertake a systematic cost-effectiveness analysis of alternative antimalarial drugs (SP, CQ and AQ) and to cost the policy change to an alternative regimen, and finally to inform the NMCP, the research community and national policy-makers. The study projected that within the next 10-year-period, the cost of transforming the new treatment policy from CQ to SP as first-line, would be half that of maintaining CQ as the first-line because the decreasing effectiveness of CQ led to recurring episodes and repeated treatment of patients. It was furthermore revealed that within the said period the cost of using SP instead of CQ would cost around US $0.46 per operational failure averted, or US $33 per death averted, considering changes in outpatient drug cost only. The study further concluded that the cost of adopting AQ would be marginally lower than that of using SP, but due to increasing incidences of side-effects, AQ should be reserved as the second-line, therefore, recommending that SP be introduced as an interim first-line drug anticipating that within the next 10-year period a better drug would be identified [6,25].

#### TANHERF

Launched in 1999 through consultative strategy facilitated by the Commission For Health Research and Development (COHRED), the Tanzania National Health Research Forum (TANHERF) is composed of partner institutions in health research and their representatives (see Figure 1) and is an inclusive body whose mission is to ensures that each partner has clear defined role, is considered as an asset and shares in the ownership of the mechanism. Its main function is to ensure that evidence based information is correctly utilized by policy-makers and health managers in order to facilitate the provision of better health services to populations. Working more closely with the NIMR where its Secretariat is based, TANHERF is a consultative body to policy and decision-makers defining health research priorities and research undertakings, coordination, collaboration, dissemination of findings and utilization of research results into policy-oriented decision-making. TANHERF receives and approves reports from the Essential National Health Research Coordinating Committee and the National Health Research Ethics Committee and is a custodian for the dissemination of all the national health research results and through its collaborating institutions, it supports health research publications including research on drug resistance and antimalarial treatment options [28]. As some of its members are also members of the previously mentioned drug task force and the NMAC, TANHERF has influenced indirectly the policy change process.

### Consensus building

Researchers and members of the Task Force recognized that numerous and sometimes conflicting evidence had been presented by researchers through technical research reports and/or presentations at scientific conferences and publications in peer reviewed journals. The issue of how to reconcile the evidence and deliver a common, simple and clear message to policy-makers was raised. The solution proposed was to organize stakeholder workshops to identify, discuss and synthesize the most pertinent research and health facility-based information concerning malaria treatment and the way forward. The NMCP in liaison with the NMAC, national Drug Policy Task Force and WHO Country office organized malaria workshops and meetings with departmental heads and directors within the MoH and other partner institutions such as the pharmaceutical industry, bednet manufacturing industries, research institutions such as NIMR, IHRDC and academic institutions such as the MUCHS, St. Augustine University Teaching Hospital – Bugando and KCMC, and intervention projects such as Tanzania Essential Health Interventions Project (TEHIP) and the Adult Morbidity and Mortality Project (AMMP). One of the latest workshops held was the one of May 1999 in Bagamoyo in Tanzania supported by the WHO Roll Back Malaria (RBM) Programme. Based on the presentations made by several participants during the workshops, the discussion focused on developing a common way of understanding the trend of drug resistance and the pros and cons of whether to maintain the status quo with CQ as the first-line drug or to switch to alternative drugs like SP, AQ, chlorproguanil-dapsone (Lapdap) or others.

Despite the intense discussion about alternative drug regimens, there was a common appreciation that CQ treatment failure rates at different levels and in different drug monitoring sentinel sites were alarming enough to justify the need for change. The remaining question that was left for Parliament to answer in liaison with the MoH and other partners (e.g. NMCP, NMAC, Drug Policy Task Force) was whether to replace CQ either with SP as a single first-line therapy or in combination with other regimens. RBM, through its representative in East Africa, expressed readiness to support technically and materially where necessary and feasible to facilitate any government policy change decision. As one of the respondents argued, this was a very important message to the country policy-makers because of the budget implications of such a change.

### The actual policy change process and current perceptions of the change

After the national Task Force on antimalarial drug policy had presented its policy brief, a series of newspapers and some private radio stations began to inform the public that CQ was no longer a recommended drug for malaria treatment and that the Government was considering replacing it with a new drug. This information caused public concern and debates erupted in different parts of the country about the rationality for the change. Those involved were the general public, the research community, traders, the pharmaceutical industry, and health-care providers in the public and private health facilities. To maintain public confidence, the Minister of Health gave out a press release that indicated the Government stand concerning the treatment guidelines to be followed while strategies were underway to make an appropriate decision. Nobody was assured of the time of the actual policy change and which type of new treatment guideline would be recommended, as a senior health manager remarked while interviewed in this study:

"It was not clear when the policy would have changed, as sometimes information that was coming out in the press releases last year was controversial. There is a time when the Minister for Health seemed to criticize the information that came out in one of the local newspapers that CQ was no longer effective, and advised the public to remain patient until his ministry gets sufficient evidence. On the other hand, researchers continued to disseminate information indicating high levels of resistance and suggesting for finding out a more suitable drug".

Also, according to several respondents, the policy change was not in the interests of the pharmaceutical manufacturers and traders, who had built up large stocks of CQ and had profited much from its familiarity among most of their client populations. Drug supply companies had already invested in small vans to deliver CQ with a banner 'CHLOROQUINE' on their sides.

Medical practitioners and biomedical researchers considered it was too early to change the policy. One of the reasons stated was that many people still believed that CQ was effective despite variations between different areas and that SP resistance was reported to be on the increase. Some high-ranking government officers at parliamentary and ministerial levels identified themselves as being among those who were still using CQ effectively. Detractors also relied on a few incidences of patients who had had side effects with other antimalarial drugs and also pointed paucity of information available regarding SP resistance. This fact is similar to one found in the report by Abdulla *et al *[6] warning that:

"Anecdotal evidence indicates that many health professionals are unaware of the extent of resistance to CQ and do not perceive an urgent need for change".

It was also expressed that the decision to change the policy was a very sensitive issue considering the financial implications of the change, both to the government and to the users of the drugs on one hand, and the lack of expertise to manage the change and the uncertainty of treatment outcomes in the use of the new drug.

The Government's official announcement of the policy change came out of the media in 2000, although the actual implementation officially started on 1^st ^August 2001. Before this Government policy-decision was passed, approval by the national parliament was sought through the speech by the Minister of Health by then to the Parliament while presenting his ministry's annual budget [6]. Scientific facts played a prominent role, as the Minister pointed out that based on the routine health facility-based morbidity and mortality statistics on malaria and biomedical research evidence, the MoH was convinced that it was high time for the government to replace CQ with a more cost-effective first-line drug that obviously could alter the existing treatment regimen as a whole. The agenda for change was presented based on a summary report by the mentioned Drug Policy Task Force, supplemented by collective information from HMIS records submitted to the MoH, drawn from all regions and policy-research related workshop/conference proceedings, describing the malaria situation.

A similar speech was the one presented by the new Minister of Health, this time Hon. Anna Abdallah during the 2002–2003 Budget Session for her ministry [27] in 2002. With these two speeches, the members of the Parliament (MPs) were convinced of the need for change, albeit the decision to switch would be interim, given that more clinical and cost-effectiveness studies were ongoing in some sentinel sites to establish more evidence regarding the most appropriate treatment option. This was justified by the presence of the 5-year Interdisciplinary Monitoring Programme for Antimalarial Drugs in Tanzania (IMPACT-Tz) whose initiation involved the MoH as a key stakeholder. The IMPACT-Tz project is a collaborative venture between the U.S Centres for Disease Control (CDC), IHRDC, NIMR, LSHTM, AMMP, TEHIP, MUCHS, WHO and district health authorities [27,30].

All the respondents in this study expressed the need for continued research on alternative drug regimens that are cost-effective if used in real situations, and to publish systematically organized policy-briefs to keep policy-makers and national programme managers informed of the malaria treatment situation and its socio-economic consequences. It was clarified that each research project should consider the presentation of concise policy feedback report by arranging to meet directly, where possible, with policy-makers and programme managers to present their findings and recommendations and share discussions with them whereby various questions can be answered on the spot and reaching agreement on the way forward. It was emphasised that the policy reports should be in the simplest possible language rather than using too technical scientific jargons which would discourage most of the non-technical scientific readers among whom are some policy-makers, planners and programme managers.

## Discussion

### Factors for the delaying or speeding up of policy change process

As this case study shows (Table 2), the process of research-to-policy change is not simple, it is lengthy and tortuous, facing numerous challenges from the initiation of the research to producing evidence for policy-makers and other key stakeholders to be convinced. Thus, research intended to build consensus with policy-makers based on the evidence it has produced would be successful depending on the following:

(i) research evidence communicated clearly and timely to policy-decision makers. It needs to be communicated in a language which can convey the message to policy-making bodies, as many cannot understand biomedical technical jargon: research reports that are too technical and too long face the risk of being discarded by the audience, who are non-professional in the field or who have limited time to read/listen the whole report due to their other official commitments or personal responsibilities;

(ii) consistence of the evidence produced from different studies on the same research topic/theme: as research evidence from different studies may be more or less overlapping or contradicting each other, policy-makers find it difficult to make explicit policy change decisions;

(iii) the scale of the study on which recommendations for policy change are based: policy-makers would be interested in research evidence drawn from a wide range of study populations and evidence drawn from different country sentinel sites are more likely to be acceptable the purpose of a nationwide policy change. Sometimes, evidence from multi-country studies within the region are preferred to small scale studies undertaken in one or several areas in the country. As is evident from the present case-study, EANMAT reports have contributed substantially to convince the Tanzanian policymakers as has been the case with the rest of East African NMCPs;

(iv) the anticipated repercussions of making a policy change decision: such a decision cannot be made in isolation of the standpoint of view of the key stakeholders, especially drug manufacturers and drug traders, as these may need to be given an assurance on issues such as stocks of drug materials or circulation, which have an implication on the profit and loss accounts of their firms;

(v) policy-makers sometimes lack confidence about the reported efficacy and safety of the recommended drugs and may use a few speculative media reports and personally observed treatment outcomes to justify their reasons for delaying a policy change. As Ralston [31] has remarked, research and its synthesis and discussion helped allay the fear of uncertainty and supported action in the face of marked opposition;

(vi) more often, the government making decisions after learning from what other countries especially the neighbouring ones have done or are considering doing: If other countries have not yet changed or are considering a change to their national drug policies in a way which is different from the researchers recommendations within the country, policy decision may take longer time to change than expected by the local evidence producers/researchers;

(vi) the production of research evidence is costly: besides the budget required for the research itself and its dissemination, the cost of implementing a nationwide policy change (e.g. production of new guidelines, training of health workers, supply of newly recommended drug to all health service delivery levels, replacement of the stocks of the previously recommended drug, advertisement/publicity of the new treatment guidelines through the media, etc.) is expensive to the provider's e.g. government's side, besides potential cost associated with health care seeking and drug utilisation by the target members of the public. Unless policy-makers are convinced to reason without doubt about how these potential constraints would be minimised or avoided, no wonder that it will take time for the recommended new treatment policy to be approved.

### Potential challenges to the next drug combination therapy change

In response to recommendation by WHO to countries facing classical antimalarials to implement artemisin-derivatives-based combination therapy [32], the Tanzanian Government through the MoH has already explicitly announced its strategy for replacing SP monotherapy with artemisinin-based combination therapy (ACT) with effect from 2006 [33]. Such a policy move faces similar challenges such as reservations/inadequate faith by the research and drug prescribing community who anticipate immediate parasite resistance to ACT mainly due to the majority poor residents potential failure to comply with the treatment schedules because of the drug cost, individual tastes and preferences driven by their perceptions of the drug with the sulpha component and availability of alternative drugs in the liberal retail market (as some people may prefer monotherapy such as AQ or other drugs). The concern about cost related barriers both to the provision and utilisation of artemisinin drug combination therapy related services have been documented by other authors [2,34-37]. There is also failure of the drug users to take the full course/dose of the recommended drug as some of them may prefer the drug taken once and for all rather than the one whose dose has to be completed by taking it several times. Another failure may be on the side of some health practitioners to administer the drug as recommended in the policy guidelines due to lack of training on the administration of the new drug or other motives driving their practices e.g. private-for-profit health care drug administrators may be driven by the preferences of alternative types of drugs by their clients or may prescribe alternative drugs which they access easily and cheaply in the market and which apparently are more profitable.

## Conclusions and recommendations

Despite taking longer than expected, the eventual decision by the Tanzanian government to revise its malaria treatment policy in 2000 and to enforce its effective implementation in 2001 marks the explicit policy decision output backed up with research evidence. Also, the commitment by the MoH to support clinical trials e.g. the IMPACT-Tz study and the one by Abdulla *et al*., can be seen as intermediate explicit policy decision points. The present case study shows that the road towards changing a nationwide drug policy is long and multifaceted. However, the ultimate decision to change the national policy depends also on evidence backed by demand-driven systematic research. As research plays a role in modifying political, technical and social values, the following is suggested: (a) need to involve key stakeholders at all stages of research to ensure it is always demand-driven (b) need to consider how and when research information should be communicated to or between different stakeholders (e.g. inviting policy-makers and the media in policy-brief workshops/meetings), as sometimes done by EANMAT, TEHIP, NIMR, COSTECH and other institutions/organizations involved in the research-to-policy partnership. As stated by Eisenberg [38], EANMAT contributed to globalize the evidence and localize the decisions, thus helping to obtain support for change (c) the imperativeness of appreciating that is unusual for all policy-makers are able to interpret research findings presented in too technical language, so a simplified version of the biomedical language is crucial for the optimal utilisation of the evidence presented (d) need for continued support to build capacity both in research, evidence presentation and in policy analysis in developing health systems (e) The importance of multi-country evidence on drug resistance to influence national policy change decision is mostly inescapable especially in countries located in the same region (e.g. EANMAT or WENMAT countries] as part of globalization, albeit it is up to the individual countries to find out the feasibility of whatever policy change decision move in the local context and localise the decisions, as some observers recommend [36].

## Abbreviations

AFRO African Region

DANIDA Danish International Development Cooperation

DFID Department for International Development (UK)

GFHR Global Forum for Health Research

IHRDC Ifakara Health Research and Development Centre

IMPACT-Tz Interdisciplinary Monitoring Programme for Antimalarial Drugs in Tanzania

KCMC Kilimanjaro Christian Medical College

LSHTM London School of Hygiene and Tropical Medicine

MUCHS Muhimbili University College of Health Sciences

NIMR National Institute for Medical Research (Tanzania)

NMCP National Malaria Control Programme

SDC Swiss Development Cooperation

STI Swiss Tropical Institute

UNDP United Nations Development Programme

UNICEF United Nations Children's Fund

USAID United States Agency for International Development

TEHIP Tanzania Essential Health Interventions Project

TDR UNICEF/World Bank/UNDP/WHO Special Programme for Research and Training in Tropical Diseases

TMA Tanzania Medical Association

TPHA Tanzania Public Health Association

WENMAT: West African Network on Monitoring Malaria Treatment

WHO World Health Organization

## Authors' contributions

GMM (a GMP PhD candidate) was the principal investigator, participated in all the case study stages from its design through research report writing and developed the first and final drafts of the manuscript. As the Manager of the AHPSR, M.A.G.B substantially provided technical advice on the design of the study protocol, data collection tools and analtytical framework, critically reviewed the case study report and furnishing it and in the writing of the manuscript.
